# Health-related quality of life changes in patients with Q-fever fatigue syndrome: a four-year follow-up study, 10 years post-infection

**DOI:** 10.1007/s11136-026-04295-9

**Published:** 2026-06-08

**Authors:** N. C. Stemerdink, S. C. M. Heemskerk, E. Hartman, M. Wesseling, P. Tieleman, A. Burdorf, J. A. Haagsma

**Affiliations:** 1 Department of Public Health, Erasmus University Medical Center Rotterdam, Dr. Molewaterplein, 403015 GD Rotterdam, The Netherlands; 2 Q-Support, ‘s Hertogenbosch, The Netherlands; 3 C-Support, ‘s Hertogenbosch, The Netherlands

**Keywords:** Q-fever fatigue syndrome, Q-fever, Quality of life, Post infectious acute syndrome

## Abstract

**Purpose:**

Q-fever can cause long-term health complications such as Q-fever Fatigue Syndrome (QFS), which may severely impact patients’ Health-Related Quality of Life (HRQoL). This study investigated change of HRQoL in QFS patients over time, and explored predictors associated with change using longitudinal data.

**Methods:**

In this prospective observational study questionnaires were administered among Dutch individuals with self-reported QFS who were registered at Q-support, a foundation that supports, advises and informs Q-fever patients. Participants completed four annual questionnaires between 2021 and 2024, including EQ-5D-5L and EQ VAS to measure HRQoL. Changes in HRQoL were categorized as “improvement”, “deterioration”, or “stable”, using an anchor-based minimal important difference approach. Multinomial logistic regression analyses identified predictors of change.

**Results:**

A total of 199 patients were included in the final analysis. At baseline, median EQ-5D-5L utility index and EQ VAS scores were 0.647 (IQR: 0.352–0.774), and 50.0 (IQR: 34.0–60.0), respectively. After four years, 37% of patients showed improvement in EQ-5D-5L utility, 30% deterioration, and 33% remained stable. Female sex and higher baseline EQ-5D-5L utility were associated with lower odds of improvement or being stable.

**Conclusion:**

More than 10 years post-infection, HRQoL remains consistently low at group level among patients with QFS, with substantial long-term variability in individual outcomes. These findings underscore the chronic nature of QFS, its long-lasting consequences, and the importance of continued monitoring of individual health trajectories. Further studies are warranted to better understand the mechanisms underlying individual differences in recovery and to inform targeted interventions for this patient population.

**Supplementary Information:**

The online version contains supplementary material available at 10.1007/s11136-026-04295-9.

## Abstract (plain language summary)

Q-fever is an illness that can lead to long-term health problems like Q-fever Fatigue Syndrome (QFS). QFS can affect how people feel and live day to day. Little is known about recovery trajectories and changes in quality of life over time, especially many years after infection. This study looks at how health-related quality of life (HRQoL) changes in people with QFS more than ten years after getting sick. QFS patients were followed for four years to see if their health got better, worse, or stayed the same. On average, the group’s HRQoL did not change much. But when looking at individuals, more than a third either improved, got worse, or stayed the same. Females were less likely to improve than males. These results show that QFS can have a lasting impact and that each person’s experience is different. This highlights the need for personalized care and ongoing monitoring.

**Supplementary Information:**

The online version contains supplementary material available at 10.1007/s11136-026-04295-9.

## Introduction

Zoonotic diseases represent an increasing threat to public health [1, 2]. Such a disease is Q-fever, caused by the bacterium *Coxiella burnetii* [3, 4]. Transmission typically occurs through inhalation of contaminated aerosolized particles. These particles usually originate from infected livestock, particularly from birth products of animals like cattle, sheep, or goats [5]. Infection does not require direct contact with farm animals, and living nearby infected farms poses a risk, as the bacteria can spread through the air. During the large Dutch Q-fever outbreak in the Netherlands (2007–2010), exposure in affected regions was therefore widespread across the general population, and not limited to individuals working in agriculture [6]. While over half of exposed individuals remain asymptomatic [7], approximately 40% develop acute Q-fever, and among these, around 20% progress to a chronic condition known as Q-fever Fatigue Syndrome (QFS) [8].

QFS is characterized primarily by severe, long-term fatigue for at least six months or more [8]. Affected individuals may experience a range of additional symptoms, including lack of concentration, memory interference, blurred vision, cognitive difficulties, and mental health problems [9]. These symptoms can endure for years, resulting in a profound impact on patient’s Health-Related Quality of Life (HRQoL) [10].

HRQoL is a multidimensional concept reflecting a patient’s physical, mental, and social well-being. It is commonly assessed using Patient-Reported Outcome Measures (PROMs), such as the EQ-5D-5L [11, 12]. Studies have shown that QFS patients report low HRQoL, high levels of fatigue and numerous health symptoms, even a decade post-infection [13–15]. Other studies have found impaired quality of life, social functioning, and mental, and physical health compared to uninfected controls [16, 17]. While previous studies highlight the burden of QFS, they do not address how HRQoL changes within individuals prospectively over time [18].

Knowledge on the long-term change of HRQoL is particularly essential for QFS, which is classified as a Post-Acute Infectious Syndrome (PAIS) due to its persistent symptoms following acute infection. These syndromes, including Post-COVID Condition, Post-treatment Lyme Disease Syndrome, and ME/CFS, are characterized by an unexplained failure to recover from an acute infection [19]. Studying the long-term trajectory of HRQoL in QFS patients is therefore crucial for understanding the impact and progression of the condition. This knowledge can inform clinical care, guide policy decisions, and ultimately improve the lives of individuals affected by this often misunderstood and underrecognized syndrome.

To our current knowledge, no studies have examined the longitudinal change of HRQoL in QFS patients more than 10 years post-infection. To gain deeper understanding of the impact of this chronic disease on patients’ lives, the present study aimed to evaluate changes in HRQoL over a four-year period, and more than 10 years post-infection, and to identify predictors associated with these changes.

## Methods

### Study design and study population

This prospective observational study was conducted in collaboration with Q-support, a Dutch foundation commissioned by the Ministry of Health, that supports, advises, and informs patients with Q-fever, and shares knowledge with healthcare professionals [20]. All individuals with self-reported QFS and who were registered at Q-support received an e-mail invitation from Q-support to participate. The invitation included detailed study information and a link to the online questionnaire, which was developed collaboratively with researchers, healthcare professionals, and QFS patients. The questionnaire contained detailed questions about the impact of QFS on patients’ health, daily functioning, and healthcare use and experience in the preceding year. All questions were mandatory to complete. Non-respondents received up to three reminders at two-week intervals. Participants were invited to complete a total of four annual questionnaires from September 2021 until November 2024 (T1 to T4). Respondents’ data were longitudinally linked across the four annual measurements using unique, pseudonymized tokens, allowing individual responses to be matched over time while preserving participant confidentiality. Participation was voluntary, and informed consent was obtained electronically prior to survey completion. The study was approved by the Medical Ethics Review Board of Erasmus MC (MEC-2021–1606), in agreement with the requirements of the Declaration of Helsinki and the STROBE guidelines. Patients were eligible for inclusion if they spoke Dutch, were registered at Q-support, and self-reported having QFS. Exclusion criteria were being < 18 years old at baseline, having no QFS diagnosis, infected < 10 years ago at baseline, or if the year of Q-fever infection or the year of QFS-diagnosis was unknown.

### Patient involvement

People with lived experience of QFS were actively involved in multiple phases of the study. During the development phase, patients and lived-experience experts affiliated with Q-support reviewed and pilot-tested draft versions of the first questionnaire to assess clarity, comprehensibility, completeness, and feasibility. During each annual measurement, participants were able to provide additional information and feedback on the questionnaire through open-ended questions. This feedback was systematically reviewed and used to refine and update subsequent versions. Each revised questionnaire was again reviewed by people with lived experience and lived-experience experts prior to use in the next questionnaire wave. Preliminary results were discussed with patients to support interpretation and to identify meaningful ways to present the findings. Healthcare professionals and other experts contributed to the development of the study design and instruments as well to ensure clinical and methodological relevance.

### Measures

#### Sociodemographic and QFS-related characteristics

Sociodemographic and QFS-related characteristics were included in the questionnaire and were collected at baseline. Sociodemographic characteristics included age, sex, education level, living situation, paid work before QFS, smoking status, and alcohol consumption. Questions on QFS-related characteristics included years since Q-fever infection, pre-existing chronic disease, and antibiotic use and hospitalization during the acute infection.

### Health-Related Quality of Life assessment

HRQoL was measured using the EQ-5D-5L questionnaire, consisting of five levels of severity in five dimensions: Mobility, Self-care, Usual activities, Pain & Discomfort, and Anxiety & Depression [21]. For all five dimensions, the participants indicated whether they experienced no problems, slight problems, moderate problems, severe problems, or extreme problems on the day of completing the questionnaire. Using the responses on the five dimensions and the Dutch value set, the EQ-5D-5L utility index was calculated [22]. Utility indices calculated with the Dutch value set can range from − 0.45 (health state considered as worst possible health state) to 1 (full health) [22]. Four single additional items were used in this study, namely: Cognition, Sleep, Tiredness and Social relationships [23–28]. These additional items are referred to as “bolt-ons”. All bolt-ons followed the EQ-5D-5L format with response options “no problem”, “slight problem”, “moderate problem”, “severe problem” or “extreme problem” with a recall period of “today”. Bolt-ons were included alongside the EQ-5D-5L to capture symptom domains that are central to QFS but may be insufficiently covered by the standard EQ-5D dimensions. These bolt-ons were used to enhance descriptive validity and interpretation of patient-reported health status. In addition, health status was measured using the vertical Visual Analogue Scale (EQ VAS), ranging from 0 (worst imaginable health) to 100 (best imaginable health) [21].

### Outcome measures

The primary outcome measure was change in EQ-5D-5L utility index, using the anchored-based Minimal Important Difference (MID) created by Cheng et al. (2024) [29]. The secondary outcome measure was change in EQ VAS, using the MID for the EQ VAS.

### Statistical analysis

Descriptive statistics were performed to summarize sociodemographic and QFS-related characteristics, as well as HRQoL outcomes. Continuous variables were reported as means (SD) and medians (IQR), and categorical variables were presented as numbers and percentages. As the utility indices were not normally distributed according to the Shapiro–Wilk test, Wilcoxon rank-sum test or Kruskal–Wallis H tests were used to test differences in utility indices between the sociodemographic and QFS-related characteristics. When the test result was significant (*p* value < 0.05), Bonferroni’s tests were used for multiple comparisons. Non-response analysis was conducted to compare the participants included in the final analysis to those lost to follow-up.

Descriptive transitions in EQ-5D-5L utility index and EQ VAS scores across all timepoints (T1 to T4) were visualized using Sankey diagrams. To examine change in HRQoL over time, two complementary analytical approaches were applied to both the EQ-5D-5L utility index and EQ VAS scores: Linear Mixed Models (LMMs) to assess overall change trajectories, and the Probability of Superiority (PS) to quantify the likelihood that a randomly selected follow-up score exceeds a randomly selected baseline score at the individual level. Changes in HRQoL were assessed using LMMs with a random intercept for each participant to account for within-subject correlation, due to repeated measures. The model was adjusted for baseline EQ-5D-5L utility index, age (18–49, 50–65 or ≥ 66 years old), sex (male, female), education level (low, intermediate, or high), living situation (married or living with partner, or living alone or one-parent household), paid work before QFS (yes or no), pre-existing chronic disease (yes or no), years since Q-fever infection (≤ 13 years or > 13 years), hospitalization during the acute Q-fever infection (yes or no), antibiotic treatment during the acute Q-fever infection (yes or no), smoking status (former, current or never) and alcohol consumption (former, current or never). Assumptions of the models were evaluated by analysis of residuals.

The anchor based MID approach for both the EQ-5D-5L utility index and EQ VAS scores were calculated using previously established regression formulas [29]:

- EQ-5D-5L utility index

$${MID}_{utility}=0.2583 - \left(0.4907* Baseline score\right)+ \left(0.2804 * {Baseline score}^{2}\right)$$

$$\Delta Utility ={Utility}_{T4}-{Utility}_{T1}$$

- EQ VAS

$${MID}_{VAS}= 36.2555 - \left(0.6444 * Baseline score\right)+ \left(0.0029 * {Baseline score}^{2}\right)$$

$$\Delta VAS={VAS}_{T4}-{VAS}_{T1}$$

In these formulas, baseline score refers to T1 measurement for either EQ-5D-5L utility index or EQ VAS. Based on the calculated MID values, changes in HRQoL were categorized into three groups: “improvement”, “deterioration”, or “stable”. Improvement was defined as: ∆ ≥ MID, deterioration was defined as: ∆ ≤ −MID, and stable was defined as: −MID < ∆ < MID. This classification was applied separately to both the EQ-5D-5L utility index and EQ VAS scores. Thereafter, within each change group, all EQ-5D dimensions and bolt-ons dimensions were analyzed and reported. MID thresholds were used solely as a descriptive benchmark to aid interpretation of group-level changes in EQ-5D-5L index scores and not to infer clinical significance or cost-effectiveness.

Furthermore, we calculated a non-parametric effect size measure, known as the Probability of Superiority (PS) [30]. PS is the probability that, within a randomly selected pair of dependent scores, the follow-up score will be larger than the score measured at the preceding timepoint, indicating an improvement in HRQoL [31]. PS was calculated between consecutive time points (T1–T2, T2–T3, T3–T4) for each dimension and bolt-on dimension, and per EQ-5D-5L Utility or VAS change group, to describe changes in HRQoL over time. For each dimension and bolt-on dimension, the number of participants with improvement was divided by the total sample size. To account for ties (participants remaining stable), we added half the number of ties in the numerator. The PS value ranges from 0 to 1 and can be interpreted as < 0.5: more patients deteriorated than improved, = 0.5: an equal number of patients improved and deteriorated, or remained stable, > 0.5: more patients improved than deteriorated [32]. The PS values for each dimension and bolt-on dimension were used as descriptive indicators of responsiveness.

To identify predictors of EQ-5D-5L Utility and EQ VAS Change Groups, univariable and multivariable multinomial logistic regression analyses were performed [33, 34]. Independent variables included baseline EQ-5D-5L utility index, age (18–49, 50–65 or ≥ 66 years old), sex (male, female), education level (low, intermediate, or high), living situation (married or living with partner, or living alone or one-parent household), paid work before QFS (yes or no), pre-existing chronic disease (yes or no), years since Q-fever infection (≤ 13 years or > 13 years), hospitalization during the acute Q-fever infection (yes or no), antibiotic treatment during the acute Q-fever infection (yes or no), smoking status (former, current or never) and alcohol consumption (former, current or never). Collinearity [variance inflation factors (VIF) < 5] of independent variables with a *p* value < 0.10 in univariable analyses was checked [35].

All statistical analyses were performed using R version 4.4.2 (R Core Team, 2021) in RStudio version 1.4.1717 (RStudio Team, 2021). A two-sided *p* value < 0.05 was considered statistically significant.

## Results

### Patient characteristics

**Fig. 1:**
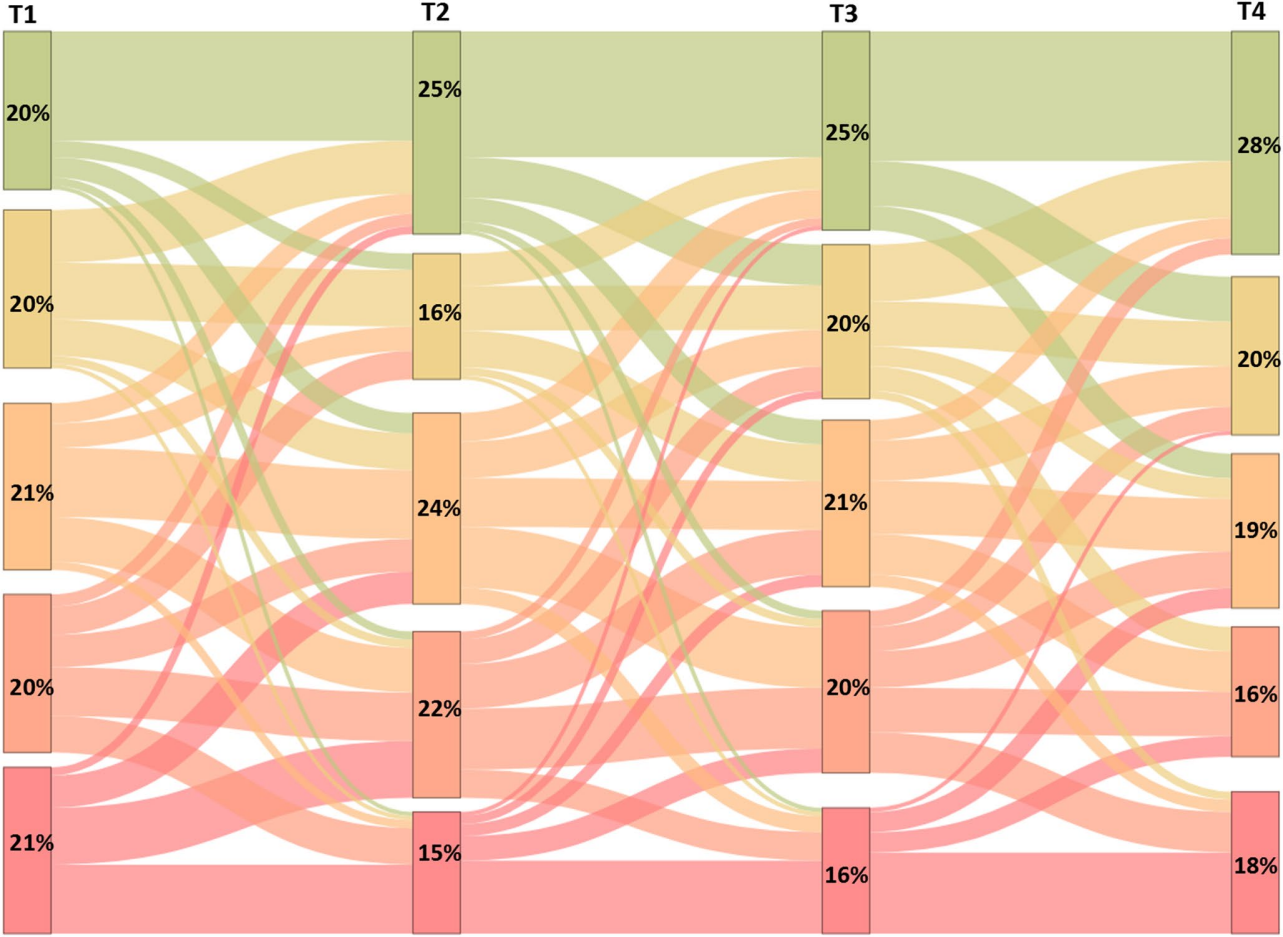
Distribution of EQ-5D-5L utility index at each timepoint (T1 to T4). *The size of the quintiles is proportional to the number of individuals within that interval. The thickness of the flows is proportional to the number of individuals within that flow. The colors correspond to the quintile distribution at baseline. From bottom to top: Q1 = − 0.34: 0.30, Q2 = 0.30: 0.58, Q3 = 0.58: 0.72, Q4 = 0.72: 0.78, Q5 = 0.78: 1.00.*

Of the 842 individuals invited to participate in the study, a total of 448 participants completed the questionnaire at baseline (T1). After excluding seven participants without QFS diagnosis, and three participants < 18 years old at baseline, 240 participants completed all four questionnaires. Sixteen participants were excluded for being infected with Q-fever < 10 years ago at baseline, and 25 participants were excluded because of unknown year of Q-fever infection or year of QFS diagnosis. Finally, 199 patients were included in the final analysis (Supplementary, Fig. 1).

The median age of the patients was 57.0 years (IQR: 50.0–64.0), and 59% were female (Table 1). The majority of the patients were married or living with partner (77%) and 41% had an intermediate education. The median time since Q-fever infection was 13.0 years (IQR: 13.0–14.0). Furthermore, 15% had been hospitalized at least once during the acute Q-fever infection. Compared to the 200 patients (50%) who were lost to follow-up after baseline measurement, those included in the final analysis were significantly more likely to be current or former drinkers (Supplementary, Table 1). In addition, no major differences in age, sex or diagnosis were observed between responders and individuals who were invited but did not participate in any of the four questionnaires.

**Table 1:** Patient characteristics and median (IQR) EQ-5D-5L utility index scores at baseline measurement

	Total N = 199 n (%)	EQ-5D-5L utility index Median (IQR)	*p* value
*Sociodemographic characteristics*			
*Sex* Male Female	82 (41) 117 (59)	0.624 (0.380–0.742) 0.679 (0.349–0.778)	0.705^†^
*Age* Mean (SD) Median (IQR)	55.1 (11.8) 57.0 (50.0–64.0)		
*Age* 18–49 years 50–65 years ≥ 66 years	49 (25) 108 (54) 42 (21)	0.657 (0.350–0.778) 0.620 (0.316–0.739) 0.721 (0.576–0.794)	< 0.05^‡^
*Education level* Low Intermediate High	52 (26) 81 (41) 66 (33)	0.639 (0.444–0.739) 0.678 (0.331–0.774) 0.647 (0.318–0.786)	0.914^‡^
*Living situation* Married or living with partner Living alone or one-parent household	154 (77) 45 (23)	0.676 (0.384–0.774) 0.596 (0.331–0.736)	0.175^†^
*Paid work before QFS* ^*a*^ Yes No	166 (83) 33 (17)	0.652 (0.349–0.774) 0.647 (0.379–0.765)	0.959^†^
*Smoking status* Never Current Former	75 (38) 35 (18) 89 (45)	0.704 (0.386–0.776) 0.542 (0.315–0.770) 0.629 (0.363–0.765)	0.228^‡^
*Alcohol consumption* Never Current Former	46 (23) 112 (56) 41 (21)	0.638 (0.288–0.766) 0.698 (0.396–0.778) 0.570 (0.320–0.739)	0.181^‡^
*QFS-related characteristics*			
*Years since Q-fever infection* Mean (SD) Median (IQR)	13.3 (1.3) 13.0 (13.0–14.0)		
*Years since Q-fever infection* ≤ 13 years > 13 years	133 (67) 66 (33)	0.682 (0.379–0.774) 0.610 (0.327–0.742)	0.199^†^
*Pre-existing chronic disease* ^*a*^ No pre-existing chronic disease ≥ 1 pre-existing chronic disease	145 (73) 54 (27)	0.695 (0.379–0.778) 0.588 (0.287–0.739)	< 0.05^†^
*Antibiotic treatment during the acute Q-fever infection* Yes No Unknown	124 (62) 62 (31) 13 (7)	0.658 (0.350–0.765) 0.686 (0.403–0.792) 0.504 (0.299–0.679)	0.109^‡^
*Hospitalization during the acute Q-fever infection* Yes No	30 (15) 169 (85)	0.702 (0.327–0.739) 0.647 (0.361–0.774)	0.779^†^
*Utility index* Mean (SD) Median (IQR)	0.564 (0.253) 0.647 (0.352–0.774)		
*EQ VAS* Mean (SD) Median (IQR)	49.4 (18.2) 50.0 (34.0–60.0)		

^a^Paid work before QFS and pre-existing chronic disease measured at T3

^†^*p* values according to the Wilcoxon rank-sum test

^‡^*p* values according to the Kruskall-Wallis H test

### Health-Related Quality of Life at baseline

The median baseline EQ-5D-5L utility index was 0.647 (IQR: 0.352–0.774), and median EQ VAS score was 50.0 (IQR: 34.0–60.0) (Table 1). The EQ-5D-5L utility index differed significantly between age and pre-existing chronic disease subgroups. The index was highest in patients aged 66 years and older (median: 0.721, IQR: 0.576–0.794), and lowest in patients 50 to 65 years old (median: 0.620, IQR: 0.316–0.739) (*p* < 0.05). Furthermore, the EQ-5D-5L utility index was higher in patients without pre-existing chronic disease (median: 0.695, IQR: 0.379–0.778), compared to patients with pre-existing chronic disease (median: 0.588, IQR: 0.287–0.739). Supplementary Table 2 shows the mean (SD) EQ-5D-5L utility index scores.

### Change in Health-Related Quality of Life over time

**Fig. 2:**
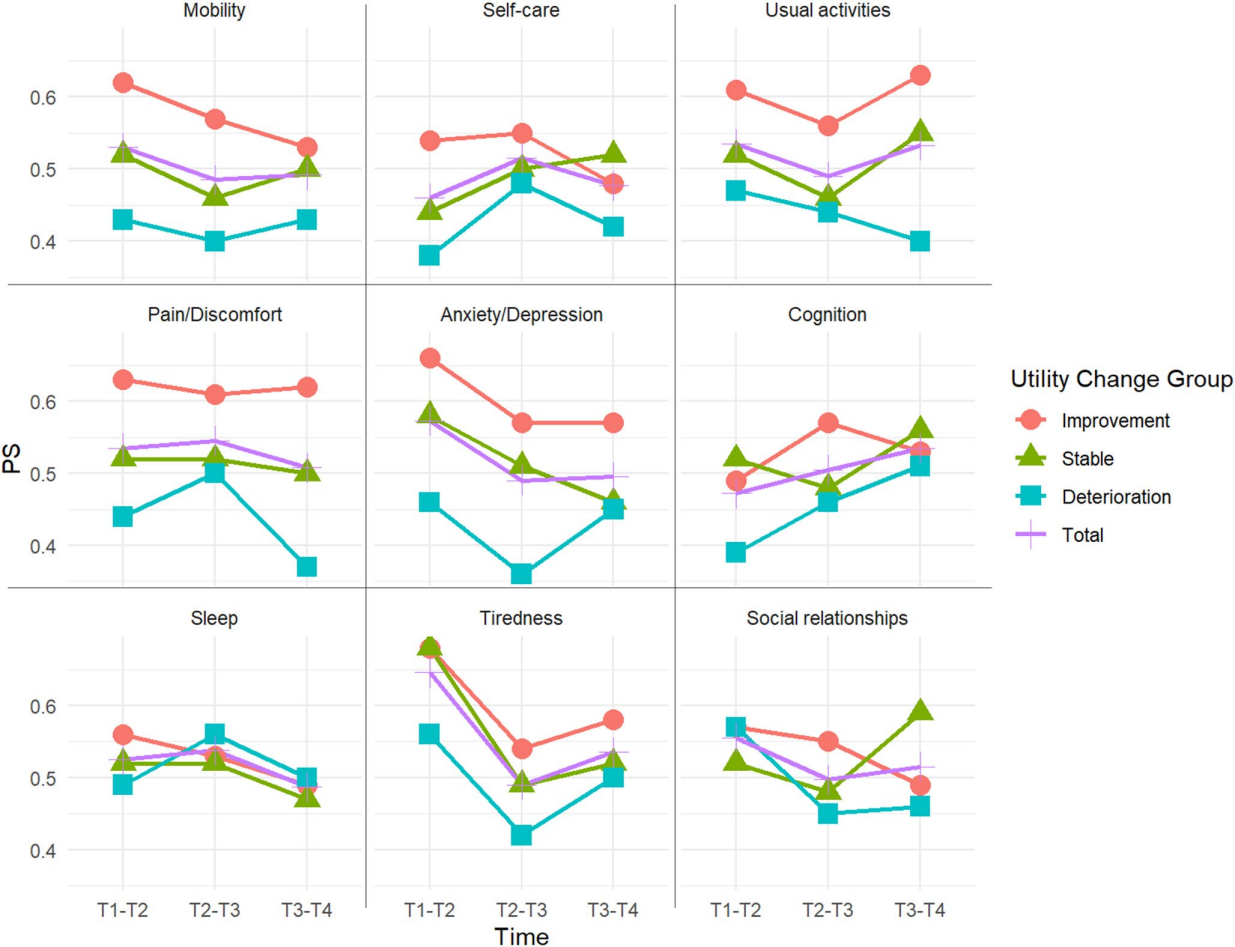
Probability of Superiority^a^ between timepoints for EQ-5D-5L dimensions and bolt-on dimensions by EQ-5D-5L Utility Change Group^b^. *PS* = probability of superiority. ^a^The PS value ranges from 0 to 1 and can be interpreted as < 0.5: more patients deteriorated than improved, = 0.5: an equal number of patients improved and deteriorated, or stable, > 0.5: more patients improved than deteriorated. Exact values are provided in Supplementary, Table 4. ^b^Anchor based Minimal Important Difference to classify as improvement, deterioration, or stable.

Figure 1 illustrates the transitions in EQ-5D-5L utility score quintiles across all timepoints (T1 to T4). The Sankey diagram reveals that some individuals improved (moving to higher quintiles), some deteriorated (moving to lower quintiles), and others remained stable (remaining in the same quintile) across timepoints. For example, among patients in the top quintile at T1, some remained there at T2, while others shifted to lower quintiles. Similar transitions are observed from lower to higher quintiles. Comparable patterns are observed in the EQ VAS Sankey diagram (Supplementary, Fig. 2).

Table 2 presents the total EQ-5D-5L utility index across four timepoints (T1 to T4) and stratified by EQ-5D-5L Utility Change Group. Among all patients, 37% (n = 74) experienced an improvement, 30% (n = 59) deterioration, and 33% (n = 66) remained stable. Within the total sample, no differences in EQ-5D-5L utility index were observed between T1–T2 and T1–T3, while significant differences were observed between T1–T4 (*p* < 0.05). An intraclass correlation coefficient (ICC) of the random effects of 0.24, indicates that 24% of the total variance was attributable to between-person differences, and 76% due to within-person fluctuations over time (results not shown). At baseline (T1), the median EQ-5D-5L utility index was lowest in the improvement group (0.52, IQR: 0.31–0.72) and highest in the deterioration group (0.72, IQR: 0.60–0.79). While, at final follow-up (T4), the deterioration group had the lowest median EQ-5D-5L utility index (0.55, IQR: 0.30–0.69), and the improvement group had the highest index (0.77, IQR: 0.67–0.75).

**Table 2:** EQ-5D-5L utility index scores at each timepoint (T1 to T4) by EQ-5D-5L Utility Change Groups^a^

	Total N = 199		Deterioration N = 59	Stable N = 66	Improvement N = 74
Timepoint	Estimated means (95% CI)	*p* value^b^	Unadjusted means (95% CI)		
T1	0.56 (0.52–0.61)		0.66 (0.61–0.71)	0.57 (0.50–0.63)	0.48 (0.43–0.54)
T2	0.59 (0.55–0.63)	0.087	0.60 (0.54–0.67)	0.57 (0.49–0.64)	0.61 (0.56–0.66)
T3	0.59 (0.55–0.64)	0.057	0.53 (0.46–0.60)	0.57 (0.51–0.64)	0.67 (0.63–0.71)
T4	0.60 (0.56–0.65)	< 0.05	0.44 (0.36–0.52)	0.58 (0.52–0.65)	0.75 (0.72–0.78)

^a^Anchor based Minimal Important Difference to classify as improvement, deterioration, or stable. EQ-5D-5L Utility Change Group values are unadjusted means (95% CI) as no separate models were fitted per subgroup.

^b^*p* values according to Linear Mixed-effects Models. Reference variable is EQ-5D-5L utility index at T1*.* Model adjusted for variables: baseline EQ-5D-5L utility index, age, sex, education level, living situation, paid work before QFS, pre-existing chronic disease, years since Q-fever infection, hospitalization during the acute Q-fever infection, antibiotic treatment during the acute Q-fever infection, smoking status and alcohol consumption.

Within the EQ VAS Change Groups, 23% (n = 47) experienced deterioration, 54% (n = 109) stability, and 21% (n = 43) improvement (Supplementary, Table 3). Similar to the EQ-5D-5L Utility Change Groups, higher EQ VAS scores at baseline were observed in the deterioration group compared to the improvement group, and at final follow-up EQ VAS scores were higher in the improvement group compared to the deterioration group.

**Table 3:** Univariable and multivariable multinominal logistic regression analyses of predictors associated with EQ-5D-5L Utility Change Groups^a^

	Univariable^b^				Multivariable^b^			
	Stable (N = 66)		Improvement (N = 74)		Stable (N = 66)		Improvement (N = 74)	
	OR (95% CI)	*p* value	OR (95% CI)	*p* value	OR (95% CI)	*p* value	OR (95% CI)	*p* value
Baseline EQ-5D-5L utility index	0.14 (0.03, 0.72)	< 0.05	0.04 (0.01, 0.21)	< 0.05	0.08 (0.01, 0.51)	< 0.05	0.02 (0.00, 0.13)	< 0.05
Sex Male (ref) Female	0.35 (0.16, 0.76)	< 0.05	0.31 (0.15, 0.66)	< 0.05	0.25 (0.10, 0.61)	< 0.05	0.24 (0.10, 0.59)	< 0.05

This table presents the results of the univariable and multivariable multinomial logistic regression analysis. The determinants were assessed at baseline (T1), reference variable is deterioration (n = 59).

*OR* odds ratio, *CI* confidence interval.

^a^Anchor based Minimal Important Difference to classify as improvement, deterioration, or stable.

^b^Both models also included the independent variables; baseline EQ-5D-5L utility index, age, sex, education level, living situation, paid work before QFS, pre-existing chronic disease, years since Q-fever infection, hospitalization during the acute Q-fever infection, antibiotic treatment during the acute Q-fever infection, smoking status and alcohol consumption.

### Probability of superiority

The results of PS across five dimensions and four bolt-on dimensions revealed change according to EQ-5D-5L Utility Change Group (Fig. 2 and Supplementary, Table 4). The patient distribution of the dimensions and bolt-on dimensions is provided in Supplementary, Fig. 3. Across all five dimensions and three bolt-on dimensions (i.e. Cognition, Tiredness and Social relationships), overall higher probabilities of change were observed within the improvement group (PS > 0.5) and lower probabilities within the deterioration group (PS < 0.5). The Sleep bolt-on dimension showed slightly higher PS in the deterioration group between T2-T3 (PS = 0.56) compared to the improvement (PS = 0.54), stable (PS = 0.52), and total (PS = 0.54) group. Similar observations were seen for the EQ VAS Change Groups (Supplementary, Fig. 4 and Table 5). Between T2 and T3, patients in the deterioration group showed higher PS compared to the improvement group for the dimensions Mobility and Social relationships. The PS of Sleep was around 0.5 for all groups at all timepoints.

### Predictors of change in Health-Related Quality of Life over time

Sex and baseline EQ-5D-5L utility index were identified as predictors of change in EQ-5D-5L Utility Change Groups in both the univariable and the multivariable model (Table 3). Higher baseline EQ-5D-5L utility index was associated with lower odds of improvement (OR 0.02, *p* < 0.05, 95% CI: (0.00, 0.13)) or being stable (OR 0.08, *p* < 0.05, 95% CI: (0.01, 0.51)). In addition, females had significantly lower odds of experiencing stability (OR 0.25, *p* < 0.05, 95% CI: (0.10, 0.61)) or improvement (OR 0.24, *p* < 0.05, 95% CI: (0.10, 0.59)) compared to males. Similarly, in the EQ VAS Change Groups, higher baseline scores were associated with lower odds of experiencing stability, or improvement (Supplementary, Table 6).

## Discussion

This prospective cohort study examined change in HRQoL in Dutch QFS patients during a four-year follow-up period, more than 10 years post-infection. Although slight differences in group level HRQoL were observed over time, substantial heterogeneity was revealed by plotting HRQoL. Additionally, when examining the EQ-5D-5L Utility Change Groups, approximately one-third of the patients showed improvement, one-third deteriorated, and one-third remained stable. In contrast, Change Groups measured by the EQ VAS resulted in a different distribution of change: more than half of the patients remained stable, while about 20% deteriorated, and 20% improved. This discrepancy may reflect the nature of the VAS, as a single-item measure of current overall health [36]. Moreover, the heterogeneous EQ-5D-5L utility index and EQ VAS trajectories observed over four years underscore the chronic and unpredictable course of QFS, with only one third of patients reporting sustained improvement in HRQoL.

Our finding that more than 75% of the total variance in HRQoL was attributable to within-person variation, highlights the heterogeneity across patients and within individuals. This emphasizes the value of frequent and longitudinal follow-up in capturing HRQoL changes, that would be missed by cross-sectional measurements [37]. Similar to a previous study in post-COVID patients, the within-person variability also supports the need to examine individual trajectories rather than just group-level changes [38].

It is important to acknowledge that some of the observed changes in HRQoL over time may be partially attributable to regression to the mean effect [39]. This occurs when patients with very high or low baseline EQ-5D-5L utility index scores tend to have scores closer to the group average at subsequent measurements, independent of any true change in their underlying health status. To minimize this effect, we included baseline EQ-5D-5L utility index as a covariate in our analyses [39–41]. The significance of the baseline EQ-5D-5L utility index in our models suggests that baseline health status influences subsequent HRQoL scores. While this adjustment helps mitigate regression to the mean, it does not fully eliminate its potential impact. Therefore, the potential influence of regression to the mean should be considered when interpreting our findings.

Additionally, covariates were measured at baseline, and most included in the model were time-independent (e.g., education level, sex), primarily capturing between-person differences. However, time-dependent variables such as smoking and alcohol consumption could theoretically contribute to within-person variation over time. Moreover, other potentially influential time-varying factors, such as physical activity, sleep quality, and received social assistance, were not included in the current model [42–44]. These factors may also play a role in explaining fluctuations in HRQoL. Future research should explore which time-dependent factors are associated with HRQoL changes over time, as this may offer additional insights into within-person fluctuations and inform personalized medical guidance.

While the multinomial logistic regression approach was used to identify baseline patient characteristics associated with change, it is important to acknowledge its limitations in the context of repeated measurements. Categorizing change into three groups treats predictors as time-independent, which may not fully capture the longitudinal dynamics of time-varying factors. Alternative methods such as generalized estimating equations (GEE), which provides population-averaged estimates of change while accounting for within-person correlation, or latent growth curve modeling (LGCM) offer complementary strengths [45]. These approaches may be valuable in future studies examining time-varying predictors or heterogeneity in individual change patterns. In the present study, however, the determinants are time-independent baseline characteristics.

Given the lack of prospective HRQoL studies in QFS patients, we compared our findings to other chronic diseases. For instance, a study in patients with Type 2 Diabetes Mellitus (T2DM) reported a greater decline in EQ-5D utility index scores compared to controls over a five-year follow-up, while changes in EQ VAS scores did not differ between groups [36]. Conversely, longitudinal analyses in patients with acute Lyme disease, with an average follow-up of 3.9 years, found improvements in HRQoL over time [46, 47]. In comparison to these studies, our findings show substantial variability in HRQoL trajectories among QFS patients, emphasizing the need for tailored healthcare [18].

Interpretation of HRQoL change is dependent on the analytical approach used [48, 49]. In this study, we applied two complementary methods: a distribution-based approach (LMM), and an anchor-based approach (using MID thresholds). Distribution-based methods are statistically robust and useful for detecting overall trends in the data, but they lack patient-centered context [29]. QFS patients often experience fluctuating symptoms and multiple health complaints. As a result, distribution-based methods may overlook clinically meaningful individual changes that are not reflected in group level statistics. Anchor-based methods, on the other hand, are more patient-centered, as they link changes in HRQoL to thresholds that are considered meaningful from the patient’s perspective [50]. This is particularly valuable in QFS, where symptoms like fatigue, pain, and cognitive dysfunction may not be fully captured by group level statistics [50]. Despite their widespread use, no consensus exists regarding the optimal method for measuring HRQoL change, with approaches varying depending on study design, outcome characteristics, and clinical context [51].

In addition to the MID to detect change among QFS patients, we applied the Paretian Classification of Health Change (PCHC; results not shown in this paper) [30]. This approach yielded consistent results with our primary analyses, reinforcing the observation that changes in HRQoL occur heterogeneously over time.

To further explore change in HRQoL, the PS captured change across individual EQ-5D-5L dimensions and bolt-on dimensions, stratified by EQ-5D-5L Utility Change Groups. Higher PS values were observed in the improved group and lower values in the deteriorated group, particularly in dimensions such as Pain & Discomfort and Usual Activities), suggesting variability in symptom trajectories between timepoints. Sleep, however, showed consistently centered PS values around 0.5 across all groups, suggesting that sleep disturbances may persist independently of HRQoL changes in QFS patients, or that sleep issues may be uniformly present throughout the cohort. Supporting the latter, a UK study reported that 65% of QFS patients continued to experience sleep issues six years post-infection [51].

These low PS scores (< 0.4) are not observed within the EQ VAS Change Groups, however, higher maximum PS values are seen, especially within the Tiredness group. The inclusion of bolt-on dimensions in the EQ-5D-5L has been widely adopted to enhance the instrument’s validity and responsiveness [11]. The results of this study underscore the added value of bolt-on dimensions in capturing symptom-specific changes [52].

Sex emerged as a significant predictor of change in HRQoL. Females had lower odds of experiencing improvement or stability compared to males, a finding observed in the EQ-5D-5L Utility Change Groups but not in EQ VAS Change Groups. Notably, females had higher baseline HRQoL scores than males, which may suggest that the observed association is partly driven by the regression to the mean effect. Furthermore, differences in health-seeking behaviors between sexes may have introduced a systematic bias in baseline HRQoL. Men with QFS may be less likely to register with a patient support organization unless more severely affected [53, 54], which may partly explain the slightly lower baseline HRQoL observed in males compared to females in our cohort. No other significant predictors were identified, suggesting that the burden of QFS may exert a broad impact across individuals, irrespective of sociodemographic or disease-related characteristics.

In addition to the heterogeneity in HRQoL change observed over time, consistently low scores in our cohort align with previous studies among QFS patients in both the Netherlands and other countries [4, 13, 16, 17]. These findings underscore the chronic nature of QFS and its long-lasting consequences, which appear to extend well beyond the acute phase of infection. Comparable impairments have been reported in other PAIS, such as ME/CFS and post-COVID condition [55–57]. The persistently low HRQoL may be attributed to both physical and psychological distress, reflected in the high prevalence and large diversity of health complaints, as well as the persistent fatigue frequently reported by patients [10, 58, 59]. This is also supported by the numerous physical and mental health complaints observed in our cohort over time [10, 13]. Additionally, dissatisfaction with the quality of care may further contribute to diminished perceived HRQoL [13, 60].

While these factors offer important psychosocial context, the persistent impairment in HRQoL observed in QFS patients may also be underpinned by biological mechanisms that remain active long after the acute infection. Chronic immune activation, neuroinflammation, and autonomic dysfunction have been proposed as potential contributors to the long-term symptom burden in QFS. Comparable mechanisms have been described in ME/CFS and post-infectious diseases [19, 61]. QFS patients with persistent symptoms have higher immune activity, including increased levels of inflammatory responses and reduced biomarkers linked to immune regulation, which are associated with fatigue and sleep disturbances [14, 62–64]. These immunological discrepancies may suggest cell-mediated immunity in QFS patients. Underlying mechanisms should be further investigated in QFS patients.

This study has several notable strengths. This is the first study to investigate HRQoL in patients with QFS more than a decade post-infection, with a follow-up period of four years. The inclusion of bolt-on dimensions to the EQ-5D-5L dimensions contributes to greater validity and provides a more comprehensive understanding of HRQoL outcomes [65]. Nevertheless, certain limitations must be acknowledged. The study population was limited to individuals with QFS who registered themselves at Q-support, potentially introducing selection bias and limiting the generalizability of the findings [66]. Second, the diagnosis of QFS was based on self-report rather than clinical confirmation, which may affect diagnostic accuracy [67]. Additionally, the extended follow-up period introduces a potential risk of attrition bias. One in four (24%; N = 199), of the 842 patients, invited to participate was included in the final analysis, reflecting a relatively low response rate. Although differences in alcohol use were observed between included participants and patients lost to follow-up, no significant differences were found in other baseline characteristics, suggesting minimal impact on internal validity. Finally, the absence of detailed information on the full QFS population in the Netherlands and internationally, combined with potential selection and attrition bias, limits the ability to evaluate the generalizability of our findings.

## Conclusion

This study highlights the importance of long-term monitoring and tailored support for QFS patients, even many years after the initial infection. The substantial long-term variability in individual outcomes, combined with the limited overall recovery in HRQoL over time, underscores the need for personalized care and support. To better understand the chronic burden of QFS, future research should focus on longitudinal assessments in larger and more diverse patient populations. Additionally, more research is needed to investigate the mechanisms underlying differential trajectories, such as sex differences, and why some patients improve while others do not. Such insights are essential for developing targeted interventions aimed at improving long-term HRQoL and overall health outcomes.

## Supplementary Information

Below is the link to the electronic supplementary material.

Supplementary Material 1

## Data Availability

There are legal restrictions on publicly sharing the de-identified data set as patient privacy cannot be fully guaranteed. This is a requirement under the General Data Protection Regulation (GDPR) and is also in accordance with Erasmus MC’s data sharing and privacy guidelines. The dataset supporting the conclusions of the current study is owned by Q-support and will be available for researchers who meet the criteria for access to data upon request. The data access request can be submitted to the Data Access Committee of Q-support (email to: info@q-support.nu).
