# Supplementary material for: Health-related quality of life changes in patients with Q-fever fatigue syndrome: a four-year follow-up study, 10 years post-infection
Source: Qual Life Res. 2026 Jun 8;35(7):191. doi: 10.1007/s11136-026-04295-9 (PMC13246837; doi:10.1007/s11136-026-04295-9)
Supplement: Supplementary file 1 — Supplementary Material 1 [file 11136_2026_4295_MOESM1_ESM.docx]

**Figures and Tables Supplementary**

**
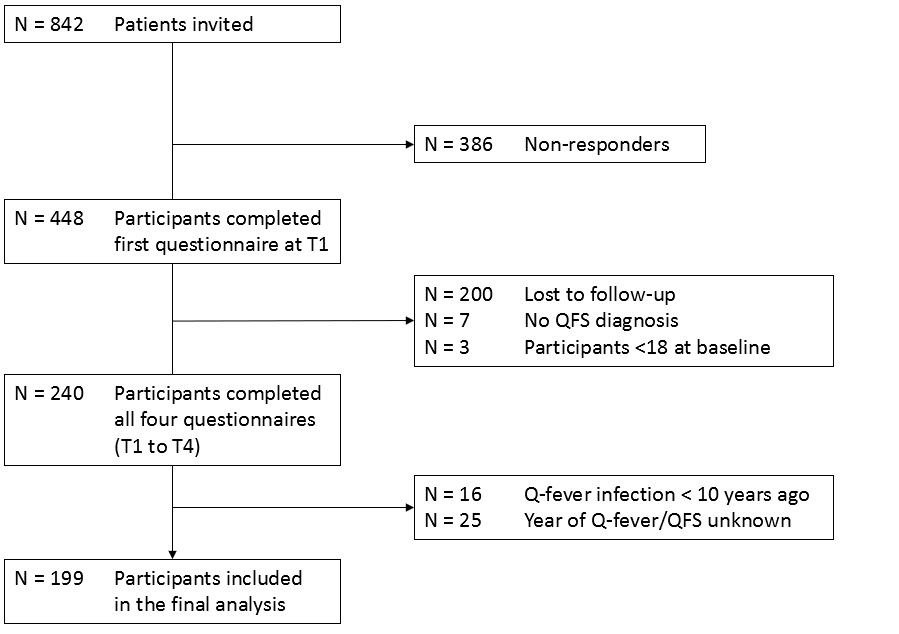

Figure 1: Participant flow diagram**

**Table 1. Patient characteristics at baseline measurement compared to those lost to follow-up**

|  | Patients included in  final analysis  N=199 n (%) | Lost to follow-up  N=200 n (%) | p*-*value |
| --- | --- | --- | --- |
| Socio-demographic characteristics | | | |
| Sex  Male  Female | 82 (41)  117 (59) | 98 (49)  102 (51) | 0.143 |
| Age  Mean (SD)  Median (IQR) | 55.1 (11.8)  57.0 (50.0-64.0) | 54.3 (13.5) 56.0 (46.0-63.3) | 0.487 |
| Age  18-49 years  50-65 years  ≥66 years | 49 (25)  108 (54)  42 (21) | 68 (34)  93 (47)  39 (20) | 0.116 |
| Education level  Low  Intermediate  High | 52 (26)  81 (41)  66 (33) | 56 (28)  84 (42)  60 (30) | 0.784 |
| Living situation  Married or living with partner  Living alone or one-parent household | 154 (77)  45 (23) | 137 (69)  63 (32) | 0.059 |
| Smoking status Never  Current  Former | 75 (38)  35 (18)  89 (45) | 87 (44)  31 (16)  82 (41) | 0.493 |
| Alcohol consumption  Never  Current  Former | 46 (23)  112 (56)  41 (21) | 71 (36)  92 (46)  37 (19) | <0.05 |
| QFS-related characteristics | | | |
| Antibiotics during the acute Q-fever infection  Yes  No  Unknown | 124 (62)  62 (31)  13 (7) | 122 (61)  67 (34)  11 (6) | 0.829 |
| Hospitalization during the acute Q-fever infection  Yes  No | 30 (15)  169 (85) | 40 (20)  160 (80) | 0.245 |
| Utility index  Mean (SD)  Median (IQR) | 0.564 (0.253) 0.647 (0.352-0.774) | 0.565 (0.26) 0.620 (0.419-0.752) | 0.960 |
| EQ-VAS  Mean (SD)  Median (IQR) | 49.4 (18.2)  50.0 (34.0-60.0) | 50.1 (21.0)  50.0 (35.0-65.0) | 0.725 |

**Table 2. Patient characteristics and mean (SD) EQ-5D-5L utility index scores at baseline measurement**

|  | Total  N=199 n (%) | EQ-5D-5L utility index Mean (SD) |
| --- | --- | --- |
| Sex  Male  Female | 82 (41)  117 (59) | 0.565 (0.234) 0.563 (0.266) |
| Age  18-49 years  50-65 years  ≥66 years | 49 (25)  108 (54)  42 (21) | 0.549 (0.264)  0.535 (0.259)  0.657 (0.203) |
| Education level  Low  Intermediate  High | 52 (26)  81 (41)  66 (33) | 0.577 (0.222)  0.557 (0.272)  0.563 (0.256) |
| Living situation  Married or living with partner  Living alone or one-parent household | 154 (77)  45 (23) | 0.578 (0.251)  0.515 (0.258) |
| Paid work before QFS^a^  Yes  No | 166 (83)  33 (17) | 0.560 (0.259)  0.582 (0.226) |
| Smoking status Never  Current  Former | 75 (38)  35 (18)  89 (45) | 0.593 (0.246)  0.499 (0.264)  0.565 (0.253) |
| Alcohol consumption  Never  Current  Former | 46 (23)  112 (56)  41 (21) | 0.549 (0.263)  0.589 (0.250)  0.511 (0.246) |
| Years since Q-fever infection  ≤13 years  >13 years | 133 (67)  66 (33) | 0.582 (0.244)  0.528 (0.268) |
| Pre-existing chronic disease^a^  No pre-existing chronic disease  ≥1 pre-existing chronic disease | 145 (73)  54 (27) | 0.587 (0.244)  0.503 (0.269) |
| Antibiotic treatment during the acute Q-fever infection  Yes  No  Unknown | 124 (62)  62 (31)  13 (7) | 0.558 (0.256)  0.598 (0.243)  0.460 (0.253) |
| Hospitalization during the acute Q-fever infection  Yes  No | 30 (15)  169 (85) | 0.553 (0.256)  0.566 (0.253) |

^a^Paid work before QFS and pre-existing chronic disease measured at T3.

**
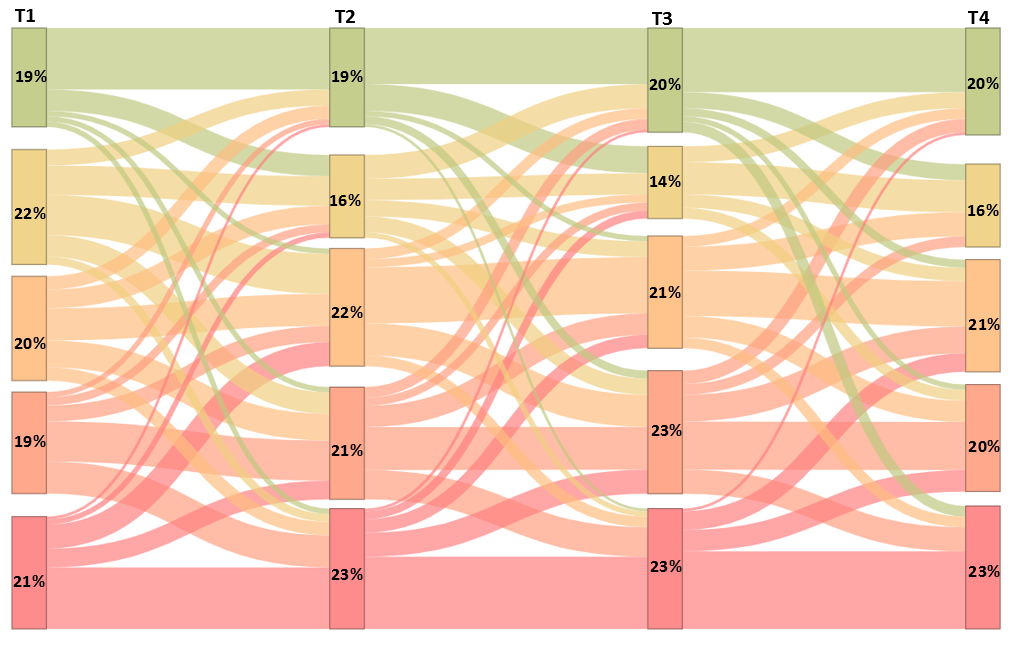
**

**Figure 2. Distribution of EQ VAS score at each timepoint (T1 to T4)***The size of the quintiles is proportional to the number of individuals within that interval. The thickness of the flows is proportional to the number of individuals within that flow. The colors correspond to the quintile distribution at baseline. From bottom to top: Q1= -0.0 : 30.0, Q2 = 30.0 : 40.4, Q3 = 40.4 : 57.8, Q4 = 57.8 : 65.0, Q5 = 65.0 : 100.0*

**Table 3. EQ VAS Scores at each timepoint (T1 to T4) by EQ VAS Change Groups^a^**

|  | **Total**  **N=199** |  | **Deterioration**  **N = 47** | | **Stable**  **N = 109** | **Improvement**  **N = 43** | |
| --- | --- | --- | --- | --- | --- | --- | --- |
| **Timepoint** | **Estimated means (CI)** | **P-value^b^** | **Mean (95% CI)** | | | | |
| **T1** | 49.4 (45.8-52.0) |  | 64.4 (60.9-67.8) | 42.5 (39.4-45.6) | | | 50.5 (45.0-55.9) |
| **T2** | 47.9 (44.4-50.6) | 0.217 | 52.7 (46.9-58.5) | 43.0 (39.8-46.2) | | | 55.1 (48.8-61.3) |
| **T3** | 47.3 (43.8-50.0) | 0.088 | 48.8 (42.8-54.8) | 42.5 (39.5-45.6) | | | 58.0 (52.3-63.6) |
| **T4** | 48.2 (44.7-50.9) | 0.336 | 41.7 (36.5-47.0) | 42.1 (38.8-45.3) | | | 70.9 (66.9-74.9) |

^a^Anchor based Minimal Important Difference to classify as improvement, deterioration, or stable. EQ VAS Change Groups values are unadjusted means (95% CI) as no separate models were fitted per subgroup.
^b^P-values according to Linear-Mixed effects Models. Reference variable is EQ VAS at T1*.*  Model adjusted for variables: baseline EQ VAS, age, sex, education level, living situation, paid work before QFS, pre-existing chronic disease, years since Q-fever infection, hospitalization during the acute Q-fever infection, smoking status and alcohol consumption. Antibiotics excluded due to n=0 in the "antibiotics = unknown" category.

**Table 4. Probability of Superiority^a^ between timepoints for dimensions and bolt-on dimensions by EQ-5D-5L Utility Change Groups^b^**

| **T1-T2** | | | | |
| --- | --- | --- | --- | --- |
|  | **Total (N=199)** | **Deterioration**  **(N=59)** | **Stable**  **(N=66)** | **Improvement**  **(N=74)** |
| Mobility | 0.530 | 0.432 | 0.515 | 0.622 |
| Self-care | 0.460 | 0.381 | 0.439 | 0.541 |
| Usual activities | 0.535 | 0.466 | 0.515 | 0.608 |
| Pain/discomfort | 0.535 | 0.441 | 0.515 | 0.628 |
| Anxiety/depression | 0.573 | 0.458 | 0.583 | 0.655 |
| Cognition | 0.472 | 0.390 | 0.523 | 0.493 |
| Sleep | 0.525 | 0.492 | 0.515 | 0.561 |
| Tiredness | 0.646 | 0.559 | 0.682 | 0.682 |
| Social relationships | 0.555 | 0.568 | 0.523 | 0.574 |
| **T2-T3** | | | | |
|  | **Total (N=199)** | **Deterioration**  **(N=59)** | **Stable**  **(N=66)** | **Improvement**  **(N=74)** |
| Mobility | 0.485 | 0.398 | 0.462 | 0.574 |
| Self-care | 0.515 | 0.483 | 0.500 | 0.554 |
| Usual activities | 0.490 | 0.441 | 0.455 | 0.561 |
| Pain/discomfort | 0.545 | 0.500 | 0.515 | 0.608 |
| Anxiety/depression | 0.490 | 0.364 | 0.508 | 0.574 |
| Cognition | 0.505 | 0.458 | 0.477 | 0.568 |
| Sleep | 0.538 | 0.559 | 0.523 | 0.534 |
| Tiredness | 0.490 | 0.424 | 0.492 | 0.541 |
| Social relationships | 0.497 | 0.449 | 0.477 | 0.554 |
| **T3-T4** | | | | |
|  | **Total (N=199)** | **Deterioration**  **(N=59)** | **Stable**  **(N=66)** | **Improvement**  **(N=74)** |
| Mobility | 0.492 | 0.432 | 0.500 | 0.534 |
| Self-care | 0.477 | 0.424 | 0.523 | 0.480 |
| Usual activities | 0.533 | 0.398 | 0.545 | 0.628 |
| Pain/discomfort | 0.508 | 0.373 | 0.500 | 0.622 |
| Anxiety/depression | 0.495 | 0.449 | 0.455 | 0.568 |
| Cognition | 0.535 | 0.508 | 0.561 | 0.534 |
| Sleep | 0.487 | 0.500 | 0.470 | 0.493 |
| Tiredness | 0.535 | 0.500 | 0.515 | 0.581 |
| Social relationships | 0.515 | 0.458 | 0.591 | 0.493 |

^a^The Probability of Superiority value ranges from 0 to 1 and can be interpreted as < 0.5: more patients deteriorated than improved, = 0.5: an equal number of patients improved and deteriorated, or stable, > 0.5: more patients improved than deteriorated.
^b^Anchor based Minimal Important Difference to classify as improvement, deterioration, or stable.

**Figure 3. Patient distribution of dimensions and bolt-on dimensions at each timepoint (T1 to T4)**

**
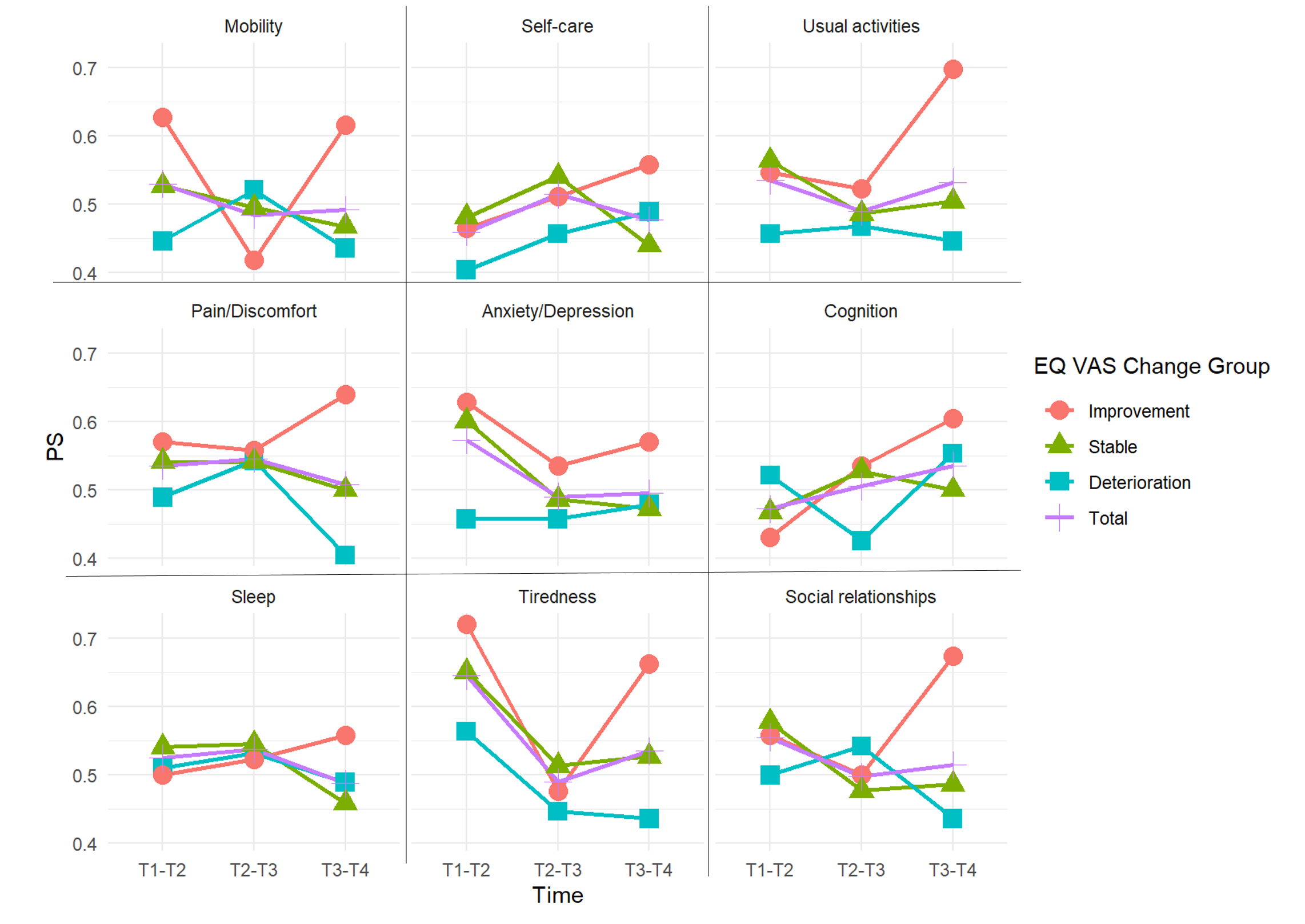
 Figure 4. Probability of Superiority^a^ between timepoints for dimensions and bolt-on dimensions EQ VAS Change Groups^b^**PS = Probability of Superiority
^a^The PS value ranges from 0 to 1 and can be interpreted as < 0.5: more patients deteriorated than improved, = 0.5: an equal number of patients improved and deteriorated, or stable,> 0.5: more patients improved than deteriorated. Exact values are provided in Supplementary, Table 4.
^b^Anchor based Minimal Important Difference to classify as improvement, deterioration, or stable.

**Table 5. Probability of Superiority^a^ between timepoints for dimensions and bolt-on dimensions by EQ VAS Change Groups^b^**

| **T1-T2** | | | | |
| --- | --- | --- | --- | --- |
|  | **Total (N=199)** | **Deterioration**  **(N=47)** | **Stable**  **(N=109)** | **Improvement**  **(N=43)** |
| Mobility | 0.530 | 0.447 | 0.528 | 0.628 |
| Self-care | 0.460 | 0.404 | 0.482 | 0.465 |
| Usual activities | 0.535 | 0.457 | 0.564 | 0.547 |
| Pain/discomfort | 0.535 | 0.489 | 0.541 | 0.570 |
| Anxiety/depression | 0.573 | 0.457 | 0.601 | 0.628 |
| Cognition | 0.472 | 0.521 | 0.468 | 0.430 |
| Sleep | 0.525 | 0.511 | 0.541 | 0.500 |
| Tiredness | 0.646 | 0.564 | 0.651 | 0.721 |
| Social relationships | 0.555 | 0.500 | 0.578 | 0.558 |
| **T2-T3** | | | | |
|  | **Total (N=199)** | **Deterioration**  **(N=47)** | **Stable**  **(N=109)** | **Improvement**  **(N=43)** |
| Mobility | 0.485 | 0.521 | 0.495 | 0.419 |
| Self-care | 0.515 | 0.457 | 0.541 | 0.512 |
| Usual activities | 0.490 | 0.468 | 0.486 | 0.523 |
| Pain/discomfort | 0.545 | 0.543 | 0.541 | 0.558 |
| Anxiety/depression | 0.490 | 0.457 | 0.486 | 0.535 |
| Cognition | 0.505 | 0.426 | 0.528 | 0.535 |
| Sleep | 0.538 | 0.532 | 0.546 | 0.523 |
| Tiredness | 0.490 | 0.447 | 0.514 | 0.477 |
| Social relationships | 0.497 | 0.543 | 0.477 | 0.500 |
| **T3-T4** | | | | |
|  | **Total (N=199)** | **Deterioration**  **(N=47)** | **Stable**  **(N=109)** | **Improvement**  **(N=43)** |
| Mobility | 0.492 | 0.436 | 0.468 | 0.616 |
| Self-care | 0.477 | 0.489 | 0.440 | 0.558 |
| Usual activities | 0.533 | 0.447 | 0.505 | 0.698 |
| Pain/discomfort | 0.508 | 0.404 | 0.500 | 0.640 |
| Anxiety/depression | 0.495 | 0.479 | 0.472 | 0.570 |
| Cognition | 0.535 | 0.553 | 0.500 | 0.605 |
| Sleep | 0.487 | 0.489 | 0.459 | 0.558 |
| Tiredness | 0.535 | 0.436 | 0.528 | 0.663 |
| Social relationships | 0.515 | 0.436 | 0.486 | 0.674 |

^a^The Probability of Superiority value ranges from 0 to 1 and can be interpreted as < 0.5: more patients deteriorated than improved, = 0.5: an equal number of patients improved and deteriorated, or stable,> 0.5: more patients improved than deteriorated.
^b^Anchor based Minimal Important Difference to classify as improvement, deterioration, or stable.

**Tabel 6. Univariable and multivariable multinominal logistic regression analyses of predictors associated with EQ VAS Change Groups^a^**

|  | Univariable^b^ | | | | Multivariable^b^ | | | |
| --- | --- | --- | --- | --- | --- | --- | --- | --- |
|  | **Stable (n=109)** | | **Improvement (n=43)** | | **Stable (n=109)** | | **Improvement (n=43)** | |
|  | **OR (95% CI)** | **p-value** | **OR (95% CI)** | **p-value** | **OR (95% CI)** | **p-value** | **OR (95% CI)** | **p-value** |
| Baseline EQ VAS | 0.92 (0.89, 0.94) | <0.05 | 0.94 (0.92, 0.89) | <0.05 | 0.90 (0.87, 0.93) | <0.05 | 0.94 (0.90, 0.97) | <0.05 |
| Sex  Male (ref) Female | 0.45 (0.21, 0.95) | <0.05 | 0.48 (0.20, 1.16) | 0.10 | 0.43 (0.16, 1.15) | 0.09 | 0.49 (0.17, 1.41) | 0.19 |

*This table presents the results of the univariable and multivariable multinomial logistic regression analysis. The determinants were assessed at baseline (T1), reference variable is deterioration (n=47).*OR = Odds Ratio; CI = confidence intervals.
^a^Anchor based Minimal Important Difference to classify as improvement, deterioration, or stable.
^b^Both models also included the independent variables; baseline EQ VAS, age, sex, education level, living situation, paid work before QFS, pre-existing chronic disease, years since Q-fever infection, hospitalization during the acute Q-fever infection, smoking status and alcohol consumption. Antibiotics excluded due to n=0 in the "antibiotics = unknown" category.
